# Optimal continuous support accompanying labor - the midwives’ and laboring women’s point of view

**DOI:** 10.1186/s13584-019-0299-3

**Published:** 2019-03-06

**Authors:** Maya Frank Wolf, Oleg Shnaider, Limor Sharabi, Sari Nahir Biderman, Reut Elon, Jacob Bornstein

**Affiliations:** 1 Department of Obstetrics & Gynecology, Galilee Medical Center, Nahariya, Israel; 2 Azrieli Faculty of Medicine, Bar-Ilan University, Safed, Israel

**Keywords:** Continuous intrapartum support, Midwives, Labor, Accompanying labor, birth

## Abstract

**Background:**

Women who have continuous intrapartum support are more likely to have a shorter labor and spontaneous vaginal birth, and are less likely to need intrapartum analgesia than women who receive usual care without support. We aimed to determine what women in labor and midwives regard as the optimal number of labor supporters and whether they should be present during medical interventions.

**Methods:**

A questionnaire was distributed to midwives participating in a national midwifery conference in June 2015. In addition, an anonymized questionnaire concerning the preferred number and type of supporters was distributed to laboring women at the beginning of labor and repeated post-partum in the maternity unit of a single tertiary medical center between March 2017 and January 2018.

**Results:**

Of 124 midwives from 18 hospitals throughout Israel attending the conference, 92 (74%) completed the questionnaire. Eighty-three percent of the midwives who responded felt that more than two supporters interferes with their work. Eighty percent of the midwives work in obstetrical units that allow up to two labor supporters, and 82% of them felt that one or two supporters is optimal. Similarly, of the 140 laboring women surveyed, 84% preferred one or two supporters. There was no difference in the preferred number of supporters between the maternal pre- and post-partum questionnaires.

The laboring women and midwives had differing opinions regarding supporter presence during vacuum extraction and perineal suture. Sixty-four percent of the midwives preferred that the supporter not be present during vacuum extraction, and 45% of them preferred that the supporter not be present during perineal suture. In contrast, among the laboring women, 78% preferred supporter presence during vacuum extraction, 76% during perineal suture and 74% during vaginal examination.

Interestingly, even among the midwives, 82% preferred that the supporter remain during vaginal examination and 84% preferred the supporter remain during medical rounds.

**Conclusions:**

Serious consideration should be given to restricting the number of labor supporters to two, as both laboring woman and midwives consider that to be the optimal number. In light of the difference of opinion regarding presence of supporters during certain medical procedures, additional surveys concerning the points of view of obstetricians and laboring women in additional hospitals should be considered before establishing a national policy.

**Electronic supplementary material:**

The online version of this article (10.1186/s13584-019-0299-3) contains supplementary material, which is available to authorized users.

## Introduction

Continuous labor support refers to non-medical assistance of laboring women and may be provided by a variety of individuals. There is some evidence that continuous one-to-one support by trained (medical staff) or untrained individuals (family, friend), provides a beneficial effect in several healthcare settings and among a variety of socioeconomic and ethnic groups, without causing any harm [1]. A 2013 Cochrane review of 16 trials involving 13,391 women concluded that women who had continuous intrapartum support were significantly more likely to have a spontaneous vaginal birth, a slightly shorter labor, and were less likely to need intrapartum analgesia or to report dissatisfaction with their childbirth experiences, compared with women who receive usual care during labor without support [1].

The main elements of labor support include attention to physical comfort (such as comforting touch, massage, and warm baths/showers), emotional support (continuous presence, reassurance and praise), explanation of procedures and assistance in making informed decisions [2, 3]. In addition, supporters provide information about labor progress and advice regarding coping techniques, and advocacy (helping the woman articulate her wishes to others). Almost all laboring women prefer having frequent or continuous support during labor to help them cope with the challenges of pain and uncertainty [4–7].

Labor support can be provided by a variety of individuals, part of the medical staff or outside to it, such as a family member, a friend or a trained doula [8, 9]. Continuous intrapartum support was found to be associated with greater benefits when the provider was not a member of the hospital staff and when the support began early in labor [1]. The midwife may teach the supporters how to encourage activities and positions known to improve the progress of labor, since labor supporters often have little experience in providing labor support [1].

The scientific basis for the beneficial effect of labor support is its effect on reduction of anxiety in labor. Anxiety during labor increases catecholamine levels, leading to unfavorable effects on labor progress and fetal outcome [10, 11]. Decreasing anxiety may have obstetrical advantages. Continuous support has been viewed as an alternative to epidural analgesia [1] and therefore could limit its effect on labor progress: it may involve less frequent use of electronic fetal monitoring, intravenous drips, synthetic oxytocin, bladder catheterization, vacuum extraction and episiotomy and carries less morbidity associated with labor and spontaneous birth [1].

However, the optimal number and type of supporters has not been addressed and many hospitals have developed their own policies regarding who and how many individuals may support a labor/delivery. Few studies have examined what is the optimal number and type of supporters accompanying labor for laboring woman and how it affects the medical staff.

Since midwives spend more time with women in labor than other medical staff, we were particularly interested in their point of view regarding the ideal number and type of supporters during labor. We were also interested in the point of view of the laboring women. Accordingly, we conducted a questionnaire survey in order to determine what laboring women and midwives regard as the optimal number of lay-support accompanying a woman during labor and whether they should be present during a variety of medical interventions.

## Methods

One hundred and twenty-four midwives from 18 medical centers throughout Israel, (7 central and 11 peripheral) attended a national midwifery conference in June 2015. The conference was not restricted to hospital based midwives. An anonymized questionnaire that does not reveal personal identifying details was distributed to the midwives during the conference, where it was completed in a group setting. The midwives’ questionnaire was composed of 11 questions: 8 dichotomous questions reflected midwives’ opinions and knowledge regarding continuous support during labor, one question about their hospital’s policy with respect to the number of supporters accompanying labor and two questions concerning what midwives regard as the optimal number of labor supporters for the laboring woman and for the midwives. The dependent variables included responses to the questions about whether laboring women should decide for themselves or whether a hospital policy be established, the midwives’ opinions concerning the presence of more than two supporters accompanying labor and whether they could interfere with their work. Other questions examined their opinion regarding the presence of the supporter during medical procedures. The independent variables included information regarding the midwives’ age, years of experience, and the characteristics of the medical centers in which they work.

In addition, an anonymized questionnaire concerning the preferred number and type of supporters was distributed to laboring women admitted at term to the maternity emergency room in a single tertiary medical center in northern Israel between March 2017 and January 2018. The questionnaire was distributed by a research coordinator midwife who explained the study goals. The laboring women who participated in the study received a similar questionnaire post-partum in the maternity department. Of the laboring women who participated in the survey 73.9% were Jews, 28.8% Muslim, 13.7% Christian, and 12.9% Druze. The questions were similar to those in the midwives’ questionnaire, including: what is the optimal number of supporters, who would they prefer as their supporters during labor and should the supporter be present during vacuum extraction, perineal suture and/or vaginal examination.

In order to compare continuous variables we used the Wilcoxon rank sum test. In order to examine the correlation between dichotomous variables we used Fisher’s exact test. An accuracy measure was calculated between dichotomous variables in order to compare the midwives’ preference with their hospital policy. As for results analysis: 60% matched answers or less was considered as coincidentally. Based on one proportional test, the one sided hypothesis, 5% significant level, for 90 responders, power was calculated as 99%. Two-sided confidence interval for 80% uniformity is 95% CI (71, 87%). These were calculated by IBM SPSS SamplePower 3.0.1.

The study was approved by the Institutional Review Board and Ethical approval of the Galilee Medical Center number 0094–15-NHR.

## Results

Approximately 600 midwives were working in Israel in 2015. One hundred and twenty-four midwives from 18 medical centers throughout Israel attended the conference. Ninety-two of them responded to the questionnaire for a response rate of 74%. The mean age of the responding midwives was 45.6 ± 9.2 and mean years of experience 13.4 ± 11.3 years. Seventy-three percent of the midwives were 40 years old or above. Of the midwives responding to these questionnaire, 80% work in obstetrical units that restrict the number of supporters during labor to two. Seventy-eight percent believed that limiting to one or two supporters is the correct policy. There was no correlation between midwives’ years of experience and their opinion concerning restricting the number of supporters accompanying labor. There was a correlation of 0.78 between midwives’ opinions for or against such restriction and their hospital’s policy to restrict the number of supporters (*p* = 0.002). Eighty-two percent of the midwives felt that as many as two supporters accompanying labor is optimal for laboring women and for the midwives as well. There was no difference in the opinions of midwives’ older and younger than 40, concerning the ideal number of supporters.

Eighty-three percent claimed that the presence of more than two supporters interferes with their work. There was no correlation between midwives’ age or years of experience and their opinion on whether an excessive number of supporters interferes with their work.

Although 84% of the midwives believe that the type of supporter should not be determined by hospital policy, in two distinct circumstances they thought it should be restricted: 69% believed that a woman’s offspring should not be her supporters accompanying labor and 54% felt that no male (i.e. father, uncle) other than the laboring woman’s partner should be in attendance. Sixty-four percent of the midwives felt that the supporter should not be present during vacuum extraction and 45% felt that the care supporter should not be present during perineal suture. Eighty-two and 84% felt that the supporter could remain present during vaginal examination or medical rounds, respectively (Table 1).

**Table 1:** Midwives perceptions concerning supporters’ presence during medical interventions

	Yes N (%)	No N (%)
Should the number of labor supporters be restricted?	69 77.5%	20 22.5%
Do excessive number of supporters disturb your work?	75 83.3%	15 16.7%
Should the supporters be present during medical intervention:		
Vacuum extraction	32 36%	57 64%
Vaginal examination	72 81.8%	16 18.2%
Perineal tear repair	49 55.1%	40 44.9%
Physician rounds	75 84.3%	14 15.7%

Concerning the survey of laboring women: 140 laboring women completed both pre and post-partum questionnaire. Maternal mean age was 29.6 ± 5.4. Thirty-eight % were nulliparous and the mean number of gestations was 2.6, of those, 10% had previous cesarean section.

In the pre-labor questionnaire 34% of the 140 laboring women preferred their partner and their mother as their supporters; 31% preferred their partner as their supporter; 8% preferred their partner, mother and mother in law; 5% preferred their mother alone; 3% preferred their partner, mother and sister. Only 3 laboring women (2%) preferred a doula as their care supporter. Of note, 3 laboring women (2%) preferred not to use labor supporters at all. 38% of the laboring women preferred one labor supporter and 46% preferred two supporters during labor; taken together this means that 84% of the women preferred one or two supporters. Only 35% of laboring women preferred their supporters to switch during labor. Seventy-eight percent of the laboring women felt that the supporter should be present during vacuum extraction, 76% preferred supporter presence during perineal suture and 74% preferred their supporter to remain present during vaginal examination.

There was no difference in the preferred number of supporters between pre and post-partum questionnaire, nor in the preference for supporter presence during vacuum extraction or vaginal examination. In retrospect, fewer post-partum women preferred their supporters to remain during perineal suture (57% vs. 76%). Postpartum, their desire to switch supporters during labor did not change. The laboring women’s expectation of their personal support changed depending on the identity of the supporter: the main expectation from personal (family) support was emotional support (73.4%) while the expectation from the midwife as a supporter included physical support (30.7%), emotional support (34.3%) and help in communication with the medical staff (8.6%). In retrospect, post-partum women felt that the main emotional support was received from their family member (64%) and the midwives were more helpful in communicating with the medical staff (70%) and lending physical support (40%). 55% of the laboring women felt that the midwives contributed more to their birth experience; 38% felt that their care supporter contributed more significantly.

## Discussion

Most of the midwives who responded to the survey believe that up to two supporters is optimal for the laboring woman, and for the midwife as well, and that an excessive number of supporters might interfere with their work. The participant midwives’ point of view concerning the number and type of labor supporters was not different from that of the laboring women in the specific institution in which the laboring women’s survey was carried out.

The majority of previous studies regarding labor supporters focused on medical benefit rather than the best way to provide the support. An excessive number of supporters accompanying labor were not previously mentioned as a barrier to continuous labor support in those studies. Laboring women claimed that positive perception of childbirth may be promoted by fewer interventions such as inductions, forceps/vacuum extraction and episiotomies, by participation in decision making and by a positive perception of supporters- partner, nurse or midwife [12]. Whether labor support should continue during vaginal examination, medical rounds or procedures such as vaginal tear suture or vacuum extraction, is not clear and could be clarified by specific guidelines in each hospital. In our study, 82 and 84% of midwives felt that the supporter should be present during vaginal examination or medical rounds, respectively.

As for vacuum extraction, 64% of midwives felt that the care supporter should not be present for these procedure. Permitting the presence of someone outside the medical staff during vacuum extraction could have some positive effects, since emotional support is a crucial element of supporting care. However, in a study of 416 nurses [13], the most frequent comment was that medical interventions such as elective labor inductions and unnecessary cesareans prevented them from providing optimal labor support to their patients.

Most of the laboring woman who participated in the survey believed that up to two supporters is optimal for them during their labor. Seventy-eight percent of the laboring women in a single institution felt that the supporter should be present during vacuum extraction, 76% preferred supporter presence during perineal suture. The existence of conflicts between the needs/wishes of the laboring women and the midwives regarding supporter presence during vacuum extraction (64% of the midwives were against and 77% of the women were in favor) and perineal suture (45% of the midwives were against and 76% of the women were in favor) may have been predictable. Most likely, the midwives prefer to clear the room of disturbances and focus on giving professional emergency assistance. The laboring women, on the other hand, probably prefer to have their care supporters present at crucial moments in the progress of the labor that could impact fetal well-being.

Perhaps the conflict could be avoided by clearly informing the birthing mother at the beginning of the labor of hospital policy concerning the presence of supporters during certain medical interventions. In this way, a conflict would not arise during the critical period of time when vacuum extraction due to fetal distress is performed. On the other hand, since other countries manage appropriate medical intervention while maintaining a woman’s support system by allowing continuous family support in the room during vacuum extraction, the Israeli medical system might adopt these practices.

In this survey, the laboring women opinion regarding the number of supporters or supporters’ presence during medical intervention did not depend on whether they were asked pre-partum or post-partum, except for their preference concerning supporters’ presence during perineal suture. Interestingly, in retrospect, only 57% of post-partum mothers preferred their supporters to remain during perineal suture (as opposed to the 76% who voiced this preference during labor) and that percentage is similar to the midwives’ wish (55%) regarding supporter presence during perineal suture. A possible explanation for the difference in women’s feelings regarding supporter presence during vacuum extraction versus perineal suture is the act of birth. Laboring women likely want their support family there for the delivery of the child, as a life cycle event; such emotions may not apply to the postpartum repair.

The comparisons between the responses of the midwives and the responses of the laboring women (regarding the optimal number of labor supporters and related issues) should be interpreted cautiously, as there are two important differences between the questionnaires administered to the two groups (which can be found in the Additional file 1). First, the laboring women were asked about their preferences for themselves during their own labor, while the midwives were asked about their views on what should be the general hospital policy. Thus, their viewpoints are complementary rather than directly comparable. Second, before they were asked about their preferences for the number of labor supporters, the laboring women were given a list of potential types of supporters (partner or husband / mother / sister / mother-in-law / friend / dula / other); the midwives were given no such list. It is possible that this may have led the midwives to consider a somewhat broader, or somewhat narrower, range of types of supporters. While this may have biased the results somewhat, our sense is that this effect is unlikely to have been large.

One limitation of this survey is the possibility that some of the survey questions may have been misunderstood by some the participants. Surveys with closed-ended (dichotomic) questions might have a lower validity rate than other question types. In addition, interviewing at a conference might bias the study towards midwives who come to a conference - perhaps older more educated ones who have leadership positions at their institutions. Perhaps another limitation of the maternal survey was that it was carried out in only one institution, although the institution serves a very diverse population of over half a million people. The limited number of women in the sample who underwent vacuum extractions might be a limitation for the particular issue of setting policy in cases of vacuum extraction.

A national policy concerning labor supporters means that there is a consistent policy that applies to each and every laboring woman in Israel, and is evidence-based on midwives’ and laboring women’s opinion and preferences. A national policy would prevent variation from one delivery room to the next, so that the laboring woman knows what to expect. At the same time, an institutional policy means that a medical center can adjust its policy to suit its specific population’s preferences. In some population sectors, the birthing mother is pressured by other family members regarding her choice of supporters, so an institutional policy may help her deflect these pressures. In the case of supporter’s presence during vacuum extraction and perineal suture, additional information regarding the obstetrician’s point of view would be helpful to complete the picture and determine a policy.

In summary, the issue concerning the number and type of labor supporters must be fully addressed, since it affects both laboring women and the medical staff. Each pregnant woman has her own personal expectations and birth plan, and that emphasizes the importance of a national medical policy on the issue of labor supporters. Additional surveys concerning the points of view of obstetricians and laboring women in additional hospitals on the issue of labor supporters including a survey targeted at women who underwent vacuum extraction, should be considered before establishing hospital policy.

## Conclusions

We recommend establishing a committee of midwives, physicians and social workers from each cultural sector in order to establish a differentiated policy that will fit each medical center patient’s population. Concerning the presence of labor supporters during medical interventions, an additional survey dealing with the obstetricians’ opinion might help in establishing policy in that issue.

## Additional file

Additional file 1: Laboring women questionnaire. Questionnaire for Midwives Concerning Labor Supporters. Post-partum questionnaire. (ZIP 42 kb)
