# Supplementary material for: Optimal continuous support accompanying labor - the midwives’ and laboring women’s point of view
Source: Isr J Health Policy Res. 2019 Mar 6;8:27. doi: 10.1186/s13584-019-0299-3 (PMC6402159; doi:10.1186/s13584-019-0299-3)
Supplement: Supplementary file 1 — Laboring women questionnaire. Questionnaire for Midwives Concerning Labor Supporters. Post-partum questionnaire. (ZIP 42 kb) [file 13584_2019_299_MOESM1_ESM.zip › Laboring women questionnaire.docx]

Date:

**Laboring women questionnaire**

Dear Parturient,

We would like to learn more about your expectations and wishes concerning your supporter/s during labor. This anonymous questionnaire will be used for a process of survey and improvement.

First part:

1. Age___
2. Years of education____
3. Religion a. Jewish, b. Muslim, c. Christian, d. Druze, e. other____
4. Degree of Faith a. secular b. traditional c. religious d. orthodox
5. Family status a. unmarried b. married c. separated d. other
6. Number of children (parity) ___
7. Gravidity (number of pregnancies) _____
8. Previous abortions Yes___ No___ number___
9. Did you participate in a preparation for childbirth course YES/NO
10. Previous cesarean section Yes / No

Second Part:

1. Would you prefer to have your labor supporter present while you wait and during fetal monitoring in the obstetric emergency room? Y/N
2. If yes who would you like to have as your supporter (you can choose more than one)
3. No-one
4. Partner
5. Mother
6. Sister
7. Mother-in-law
8. Friend
9. Doula
10. Other
11. Would you like your supporter to be present during the obstetrician's examination in the obstetric emergency room? YES/NO
12. If yes , who would you like to have as your supporter (you can choose more than one):
13. No-one
14. Partner
15. Mother
16. Sister
17. Mother-in-law
18. Friend
19. Doula
20. Other
21. Would you like to have a supporter/s during your labor? YES/NO
22. Who would you prefer as your labor supporter/s

(you can choose more than one):

1. No-one
2. Partner
3. Mother
4. Sister
5. Mother-in-law
6. Friend
7. Doula
8. Other
9. How many supporters would you prefer during your labor___
10. Would you like to switch you supporter during labor YES/NO
11. Would you like your supporter to be present during vaginal examinations YES/NO
12. If vacuum extraction is necessary, would you like your supporter to be present during this procedure YES/NO
13. If perineal tear suture is necessary, would you like your supporter to be present during this procedure? YES/NO

Third part:

1. What is in your opinion the most important element of labor support you expect from the supporter:
2. Emotional support
3. Physical support in positions
4. Maintaining privacy
5. Help with communicating with medical staff
6. What is in your opinion the most important element of labor support you expect from your midwife:
7. Emotional support
8. Physical support in positions
9. Maintaining privacy
10. Help with communicating with medical staff

Thank you for your assistance !
