# Supplementary material for: Optimal continuous support accompanying labor - the midwives’ and laboring women’s point of view
Source: Isr J Health Policy Res. 2019 Mar 6;8:27. doi: 10.1186/s13584-019-0299-3 (PMC6402159; doi:10.1186/s13584-019-0299-3)
Supplement: Supplementary file 1 — Laboring women questionnaire. Questionnaire for Midwives Concerning Labor Supporters. Post-partum questionnaire. (ZIP 42 kb) [file 13584_2019_299_MOESM1_ESM.zip › Post-partum questionnaire.docx]

Date:

**Post-partum** **questionnaire**

Mazal tov!

This part of the questionnaire refers to the experience of lobor supporting:

1. Was your supporter present while waiting and during the monitor at the obstetric emergency room? Y/N
2. If yes who?
3. Partner
4. Mother
5. Sister
6. Mother-in-law
7. Friend
8. Doula
9. Other
10. If no, why? _____
11. Was yor supporter present during during the obstetrician examination at the obstetric emergency room? YES/NO
12. If yes, who ?
13. Partner
14. Mother
15. Sister
16. Mother-in-law
17. Friend
18. Doula
19. Other
20. If no, why?________
21. Who was your supporter/s during labor?
22. No-one
23. Partner
24. Mother
25. Sister
26. Mother-in-law
27. Friend
28. Doula
29. Other
30. If no one please explain why?__________
31. In retrospect: what is the ideal number of supporeters during labor
32. In retrispect would you like your supporters to swich during labor?
33. In retrospect would you prefer yor supporter present during vaginal examination? Yes/no
34. In retrospect would you prefer yor supporter present during vacuum extraction? Yes/no
35. In retrospect would you prefer yor supporter present during perineal tear repair? Yes/no
36. In retrospect What is the most important element of labor support from your supporter?:
37. Emotional support
38. Physical support in positions
39. Maintaining privacy
40. Help with communicating with medical staff
41. In retrospect What is the most important element of labor support from your midwife?:
42. Emotional support
43. Physical support in positions
44. Maintaining privacy
45. Help with communicating the medical staff
46. Who supported you more significantly?
47. Your labor-supporter/s
48. the midwife
49. who help you more significantly with communicating the medical staff?
50. Your labor-supporter/s
51. the midwife
52. what is the most important element effecting your birth experience?
53. Admission conditions
54. professional obstetricians
55. professional midwife
56. labor supporters
57. Did the midwife lessen your need for labor supporters? Yes/no
58. What would you change concerning labor supporter accompanying labor? _______

Thank you for your assistance!
