# Supplementary material for: Optimal continuous support accompanying labor - the midwives’ and laboring women’s point of view
Source: Isr J Health Policy Res. 2019 Mar 6;8:27. doi: 10.1186/s13584-019-0299-3 (PMC6402159; doi:10.1186/s13584-019-0299-3)
Supplement: Supplementary file 1 — Laboring women questionnaire. Questionnaire for Midwives Concerning Labor Supporters. Post-partum questionnaire. (ZIP 42 kb) [file 13584_2019_299_MOESM1_ESM.zip › Questionnaire for Midwives Concerning Labor Supporters.docx]

**Questionnaire for Midwives Concerning Labor Supporters**

Part I: Demographics

1. Your age ____________
2. # years working as a midwife ___________
3. # of children you have ______________
4. Your place of work (not required) ___________________________

Part II: Please place a √ where the answer expresses your views and knowledge

|  | Yes | No |
| --- | --- | --- |
| 1, In your workplace, is there a limit to the number of people supporting a birthing mother during labor? |  |  |
| 2. If there is such a limitation, what is it? |  |  |
| 3. Do you think the number of supporters should be limited? |  |  |
| 4. Should there be a directive of who may accompany a birthing mother during labor? | Yes | No, leave it up to the mother |
| 5. Should a birthing mother's children be allowed to be present (if the mother so desires)? |  |  |
| 6. Should a man who is not the birthing mother's partner (father, brother, etc.) be allowed to be present during the labor (if the birthing mother so desires)? |  |  |
| 7. Does your workplace allow a man who is not the birthing mother's partner to be present during labor? |  |  |
| 8. Does a large number of supporters make your work more difficult? |  |  |
| 9. Should labor supporters be removed from the room during: |  |  |
| 1. Vacuum birth procedure |  |  |
| 1. PV exam |  |  |
| 1. Suturing of the perineum |  |  |
| 1. Doctors' visit |  |  |
| 10. What do you think is the ideal number of supporters for the birthing mother? |  |  |
| 11. What do you think is the ideal number of supporters for you, the midwife? |  |  |
